# Nitrogen Regulates the Distribution of Antibiotic Resistance Genes in the Soil–Vegetable System

**DOI:** 10.3389/fmicb.2022.848750

**Published:** 2022-03-14

**Authors:** Tingting Wang, Silu Sun, Yanxing Xu, Michael Gatheru Waigi, Emmanuel Stephen Odinga, Galina K. Vasilyeva, Yanzheng Gao, Xiaojie Hu

**Affiliations:** ^1^Institute of Organic Contaminant Control and Soil Remediation, College of Resources and Environmental Sciences, Nanjing Agricultural University, Nanjing, China; ^2^Institute of Physicochemical and Biological Problems in Soil Science, Russian Academy of Sciences, Pushchino, Russia

**Keywords:** antibiotic resistance genes, ammonium nitrogen, nitrate nitrogen, soil-vegetable system, transmission

## Abstract

The increasing antibiotic resistance genes (ARGs) in fertilizer-amended soils can potentially enter food chains through their transfer in a soil–vegetable system, thus, posing threats to human health. As nitrogen is an essential nutrient in agricultural production, the effect of nitrogen (in the forms NH_4_^+^-N and NO_3_^−^-N) on the distribution of ARGs (*blaTEM-1*, *sul1, cmlA, str*, and *tetO*) and a mobile genetic element (MGE; *tnpA-4*) in a soil–Chinese cabbage system was investigated. Not all the tested genes could transfer from soil to vegetable. For transferable ones (*blaTEM-1*, *sul1*, and *tnpA-4*), nitrogen application influenced their abundances in soil and vegetable but did not impact their distribution patterns (i.e., preference to either leaf or root tissues). For ARGs in soil, effects of nitrogen on their abundances varied over time, and the positive effect of NH_4_^+^-N was more significant than that of NO_3_^−^-N. The ARG accumulation to vegetables was affected by nitrogen application, and the nitrogen form was no longer a key influencing factor. In most cases, ARGs were found to prefer being enriched in roots, and nitrogen application may slightly affect their migration from root to leaf. The calculated estimated human intake values indicated that both children and adults could intake 10^6^–10^7^ copies of ARGs per day from Chinese cabbage consumption, and nitrogen application affected ARG intake to varying degrees. These results provided a new understanding of ARG distribution in vegetables under the agronomic measures such as nitrogen application, which may offer knowledge for healthy vegetable cultivation in future.

## Introduction

Antibiotics play a vital role in protecting human health and promoting the development of animal husbandry. However, the overuse and abuse of antibiotics have caused increases in resistant bacteria and antibiotic resistance genes (ARGs) in the environments (Zhu et al., 2017a; Guo et al., 2018; Zeng et al., 2019; Sun et al., 2020a; Yue et al., 2021). ARGs have been widely regarded as an emerging contaminant (Pruden et al., 2006), which would remain, transfer, and spread in different environmental media. Soil is known as an ARG reservoir, and ARGs in soil have been found to be transferred to vegetable endophytes (Yang et al., 2018), which might then enter into the human body during vegetable consumption, thus, threatening human health (Zhu et al., 2017b).

It is worth noting that some agricultural management measures could regulate ARG distribution in a soil–vegetable system. Under the application of organic fertilizers (such as manure), ARGs in the soil and the fertilizers would migrate into the plant, thus facilitating the ARG contamination (He et al., 2016). For example, Zhou et al. (2019) reported that the total ARG abundances in phyllosphere of rice was enhanced by ~20% and ~40% after a short term (within 1 month) of fertilization of pig manure collected from farms using reduced and standard antibiotic practices, respectively. Chen et al. (2018) discovered that a long term (over 10 years) of chicken manure application to soil caused a 2,638-fold ARG enrichment in the phyllosphere of maize. For inorganic fertilizers, even though they do not contain exogenous ARGs as the organic fertilizers, they could still alter ARG abundance in soil through changing the bacterial community composition (Forsberg et al., 2014). It was reported that addition of 100–200 mg kg^−1^ nitrogen fertilizer (ammonium and nitrate nitrogen) significantly increased the relative abundance of ARGs in soil (Sun et al., 2020b). As one of the typical inorganic fertilizers, nitrogen is an essential nutrient for plant growth, and applied widely and heavily in agricultural production (74 kg year^−1^ hectare^−1^ in the world; Cui et al., 2018). However, until now, studies have merely focused on their regulation on ARGs in the soil, and instead ignored their influence on the distribution and abundances of ARGs in soil–vegetable system.

The transfer of ARGs in soil–vegetable systems is a complex process. Bacteria have been reported could drive the transmission of ARGs from soil to plants *via* internal pathways (Cerqueira et al., 2019a; Zhang et al., 2019). Numerous evidences manifest that the rhizosphere is a hotspot for the spatial transmission of antibiotic resistant bacteria (ARB) from soil to vegetable and also for the horizontal transfer of ARGs from soil microbiome to vegetable endophytes (Wang et al., 2015; Yang et al., 2018). For the spatial transmission of ARB, the bacterium in the soil could secrete cell wall degrading enzymes to break through root barrier and enter the plant. They may also enter plant through natural wounds on the root surface or following the root infection (Chi et al., 2005; Liu et al., 2017; Wang et al., 2021). For gene exchanges between soil and endophytic bacteria, ARGs on mobile genetic elements (MGEs) may transfer inside of plant when soil bacteria and vegetable endophytes closely contact with each other in the rhizosphere (Barlow, 2009).

To this end, this study examined the impact of nitrogen (NH_4_^+^-N and NO_3_^−^-N) on the distribution of ARGs in the soil–Chinese cabbage (*Brassica chinensis* L.) system. The ARG abundances in soil, vegetable roots and vegetable leaves were determined by real-time quantitative polymerase chain reaction (RT-qPCR). Bioaccumulation factor (BAF) and transfer factor (TF) was determined to examine the enrichment and transferable ability of ARGs in Chinese cabbage. We further estimated the daily intake of ARGs (EI_ARGs_) to determine the exposure of ARGs to human after consumption of Chinese cabbage. This study may provide new insight to the distribution of ARGs in the soil–vegetable system under nitrogen application and improve our understanding on antibiotic resistance risks in agricultural production.

## Materials and Methods

### Experimental Design

Soil used in the pot experiment was collected from a dairy farm (31°53′6′′N, 119°15′30′′E) in Jurong, Jiangsu Province, China. The soil is a typical yellow-brown earth with the pH value of 6.6 and the moisture content of 27.5%, which contains 21.8 g kg^−1^ of organic matter. Other physicochemical properties of the soil were described in Supplementary Table S1. The soil samples were used for pot experiments after passing through a 2-mm sieve. Seven treatments were set up according to the nitrogen application level in the pots: no nitrogen (CK), 25 mg kg^−1^ ammonium nitrogen (LA), 100 mg kg^−1^ ammonium nitrogen (MA), 200 mg kg^−1^ ammonium nitrogen (HA), 25 mg kg^−1^ nitrate nitrogen (LN), 100 mg kg^−1^ nitrate nitrogen (MN), and 200 mg kg^−1^ nitrate nitrogen (HN) were added to the potting soil, respectively. The NH_4_Cl and NaNO_3_ were applied representing the ammonium nitrogen and nitrate nitrogen, respectively. Chinese cabbage seeds were soaked overnight and planted in the soil mixed with amendments. Deionization water was sprayed into the soil to keep the soil water content at 70% of the field water holding capacity. The potted vegetables were managed in the greenhouse and the soil water content was controlled by daily watering. The pots were arranged in the greenhouse with a temperature of 25 ± 2°C in daylight (12 h) and 15 ± 2°C at night (12 h) and humidity of 60 ± 5%. All treatments were conducted in triplicate. Photos of vegetable growth at 15 days (seedling period), 35 days (growing period), and 60 days (maturing period) under different treatments were shown in Supplementary Figure S1.

### Soil and Vegetable Sampling

Soil was sampled at 15, 35, and 60 days. A medicine spoon sterilized with 70% ethanol was used to collect the soil from each pot. The soil samples were stored at −80°C before the procedures of DNA extraction and chemical analysis. The vegetable samples were harvested at 15, 35, and 60 days, washed with running sterile water to remove their surface attaching soil and debris, and then divided into roots and leaves. All vegetable samples were stored at −80°C.

### Extraction of DNA From Soil and Vegetables

For soil samples, the DNA was extracted by a DNeasy Power Soil kit (Qiagen, Germany). For vegetable samples, DNA was extracted according to the method in a previous study (Chen et al., 2018), with some modifications. Around 0.5 g of the surface-disinfected vegetable sample was put into a sterile mortar. After that, the liquid nitrogen was added to the mortar, followed by an immediate trituration to vegetable samples. Then, the sample was suspended with 10 ml of sterile phosphate buffered saline (PBS). The DNA was extracted from the sample suspensions by applying the FastDNA Spin Kit for Soil (MP Biomedicals, LLC, Solon, OH, United States). The concentrations of DNA extracted from soil and vegetables were measured by Nanodrop 2000 spectrophotometer (Thermo, Waltham, MA, United States).

### Detection and Quantification of Target Genes

Five ARGs and a MGE were selected as the target genes: *β*-lactam resistance gene (*blaTEM-1*), chloramphenicol resistance gene (*cmlA*), aminoglycoside resistance gene (*str*), sulfonamide resistance gene (*sul1*), tetracycline resistance gene (*tetO*), and IS6 transposase (*tnpA-4*). The six genes had relatively higher abundances in the collected soil samples (according to the pre-test study) and were in frequent detection in the farmland soil as reported in previous studies (Knapp et al., 2010; Luo et al., 2010; Chen et al., 2019; Sun et al., 2019). Their background abundances in soil samples were shown in Supplementary Table S2. The abundance of 16S rRNA gene was also determined to quantitatively estimate the abundance of bacteria. The ARG abundances in soil and vegetable samples were determined through QuantStudio 3 Real-Time PCR System with AceQ qPCR SYBR Green Master Mix (Applied Biosystems, Foster City, CA, United States). The reaction mixture (20 μl) consisted of 10 μl of SYBR Green Master Mix (Vazyme, Nanjing, China), 0.8 μl of each primer at 10 mM (Supplementary Table S3), 1 μl of DNA template, and 7.4 μl of sterile double-distilled water (Supplementary Table S4). The reaction conditions are described in Supplementary Table S5.

### Calculation of Bioaccumulation Factors and Transfer Factors

Bioaccumulation factor (BAF) was used to quantify the ARG enrichment capacity of roots and leaves of Chinese cabbage. Transfer factor (TF) was used to examine the transfer ability of ARGs from Chinese cabbage roots to leaves.


(1) $$\mathrm{BAF}={\mathrm{RA}}_{\mathrm{cabbage}}/{\mathrm{RA}}_{\mathrm{soil}}$$

(2) $$\mathrm{TF}={\mathrm{RA}}_{\mathrm{leaf}}/{\mathrm{RA}}_{\mathrm{root}}$$

where RA_cabbage_ is the relative abundance of ARGs in Chinese cabbage roots or leaves, RA_leaf_ is the relative abundance of ARGs in Chinese cabbage leaves, RA_root_ is the relative abundance of ARGs in Chinese cabbage roots, RA_soil_ is the relative abundance of ARGs in soil.

### Calculation of Estimated Daily Intake

The estimated daily intake of ARGs (EI_ARGs_) was used to determine the copy number of ARGs consumed by eating Chinese cabbage. The daily consumption of vegetables is about 276 and 228 g (of which 15% were Chinese cabbage) for adult and child, respectively (Wang et al., 2018; Mei et al., 2021).


(3) $${\mathrm{EI}}_{\mathrm{ARGs}}=\mathrm{GCN}/g\times W_{\mathrm{intake}}\times R_{\mathrm{cabbage}}$$

where GCN/g is gene copy number of ARGs per gram of Chinese cabbage, *W*_intake_ is the weight of vegetable daily intake, *R*_cabbage_ is the rate of Chinese cabbage in raw vegetables.

### Data Analysis

The quantitative data of soil and vegetable ARGs were exported by QuantStudio Design & Analysis Software. Statistical analyses were performed using Microsoft Excel 2019 and IBM SPSS statistics 25. Differences in the gene abundances were assessed using Duncan multiple comparisons (*p* < 0.05). Data visualization were performed using Origin 2019.

## Results and Discussions

### Not All the ARGs Can Be Transferred From Soil to Vegetables

**Figure 1:**
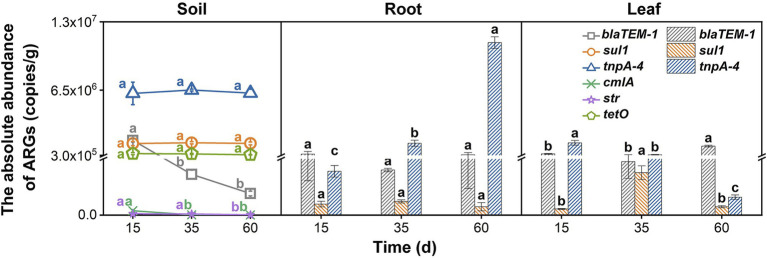
The absolute abundance of ARGs in the soil, roots, and leaves at three growth periods (15, 35, and 60 days) with no nitrogen application (CK). Error bars represent standard deviations of triplicate samples. Different lowercase letters above the bars indicated significant differences among growth periods at *p* < 0.05.

All the tested genes including the five ARGs (*blaTEM-1*, *cmlA*, *str*, *sul1*, and *tetO*) and the MGE (*tnpA-4*) could be detected in the soil. Among them, only two types of ARGs (*blaTEM-1 and sul1*) and the *tnpA-4* gene could be detected in Chinese cabbage (Figure 1), whose abundances in soil were relatively higher. This suggests that some ARGs in soil could not be transferred into vegetables. A previous study also found that only 60% of ARGs in soil were plant-transferable, and the ARG abundances decreased gradually from soil to plant (Cerqueira et al., 2019b; Xiao et al., 2021). ARGs in soil could transfer to vegetable through two major ways and the rhizosphere is a hotspot for their transfer (Van Elsas et al., 2003; Wang et al., 2015; Yang et al., 2018). Through rhizosphere some soil bacteria that carrying ARGs (i.e., ARB) could get into the vegetable and thus bring the ARGs from soil to the inside of vegetables. After entering vegetables, some ARB could grow well and the abundance of their carrying ARGs would thus increase. However, some other ARB cannot compete with the native plant endophytes and their growth would be largely suppressed, which might thus inhibit the propagation of their harboring ARGs (Xu et al., 2021). This might be a reason for the unsuccessful transfer of some ARGs (e.g., *cmlA*, *str*, and *tetO* in this study). In addition, rhizosphere is also regarded as a hotspot for gene exchange between the soil and plant microbiome (Chen et al., 2017). That is, soil bacteria harbored ARGs could transfer to vegetable endophytes by horizontal gene transfer (HGT) in the vegetable rhizosphere. In this study, the *tnpA-4* gene (a MGE) had high abundances in both soil and vegetable samples, indicating that HGT also contributed to the transmission of ARGs from soil to vegetables (Heuer et al., 2011).

### The Abundances of ARGs and the MGE in Soil and Vegetable

**Figure 2:**
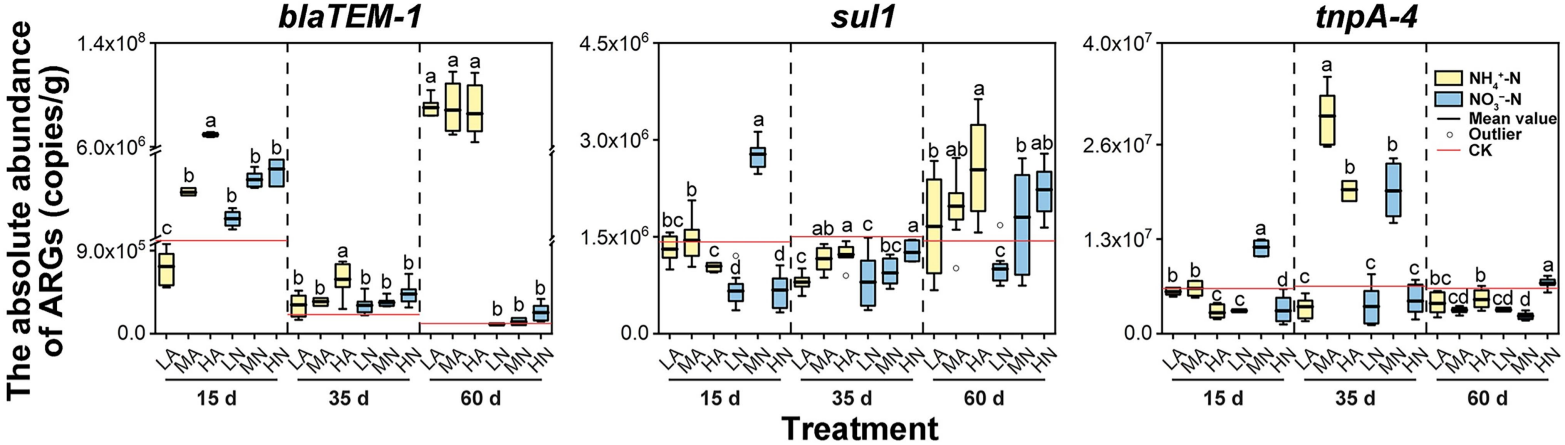
The absolute abundance of ARGs (*blaTEM-1*, *sull*, and *tnpA-4*) in the soil at three periods (15, 35, and 60 days) with nitrogen treatments: 25 mg kg^−1^ NH_4_^+^-N (LA), 100 mg kg^−1^ NH_4_^+^-N (MA), 200 mg kg^−1^ NH_4_^+^-N (HA), 25 mg kg^−1^ NO_3_^−^-N (LN), 100 mg kg^−1^ NO_3_^−^-N (MN), and 200 mg kg^−1^ NO_3_^−^-N (HN). Errors bars represent standard deviations of triplicate samples. Different lowercase letters above indicated significant among treatments at *p* < 0.05.

Nitrogen application greatly influenced the abundances of ARGs and the MGE in soil. But the influencing effect varied over time. For *sul1*, at 35 days, nitrogen obviously inhibited its abundance, which was only 53%–84% of that under the control treatment. However, at 60 days, the abundances of *sul1* with nitrogen application (particularly NH_4_^+^-N) was increased 16%–77% than the control treatment (Figure 2). On the contrary, for *tnpA-4*, its abundance reached the highest under MA, HA, LN, and MN treatments at 35 days, which increased by 202%–360% as compared with the control treatment. However, with cultivation time going (at 60 days), *tnpA-4* abundances in these treatments significantly decreased to the level similar to the control treatment (Figure 2). Previous study reported that microbial community in soil would give a quick response to the change of their growing environment (such as the change of nitrogen concentration; Geisseler and Scow, 2014), thus resulting in a better or worse growth of some population. But this alteration of microbial community was not stable at this stage (e.g., <40 days; Stark et al., 2007; Fierer et al., 2012). A period of 60 days was sufficient to stabilize the soil microbial community structure (Stark et al., 2007). This may offer an explanation for the changing influencing effect over time, and also illustrated that the results at 60 days might obtain more reference values in the practical agricultural activities. Furthermore, we particularly compared the differences in the effects on ARG abundances in soil between the NH_4_^+^-N and NO_3_^−^-N at 60 days. Obviously, NH_4_^+^-N had a more significant positive effect on soil ARGs than NO_3_^−^-N (Figure 2). It is known that NH_4_^+^-N is typically used as a primary nitrogen source for soil bacteria (Rahman et al., 2018). Therefore, soil bacteria including those with abundances positively correlated to the ARG abundances (e.g., *Nitrosospira* and *Nitrosomonas*), usually grow better under the NH_4_^+^-N exposure than under that of NO_3_^−^-N (Sun et al., 2020b, 2021). The better propagation of these soil bacteria would correspondingly increase the abundances of associated ARGs. Therefore, the promoting effect of NH_4_^+^-N on ARG abundance was stronger than NO_3_^−^-N.

**Figure 3:**
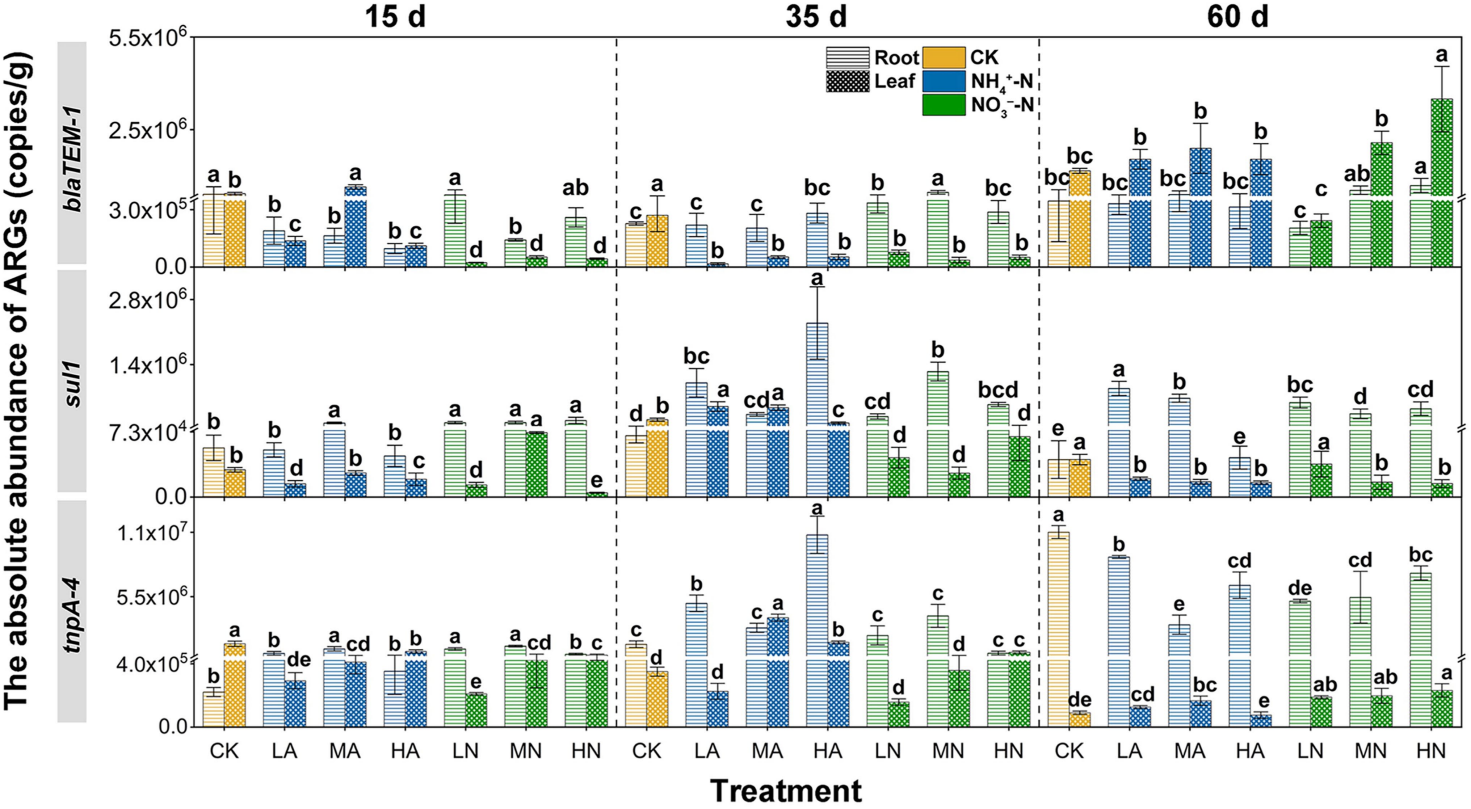
The absolute abundance of ARGs (*blaTEM-1*, *sull*, and *tnpA-4*) in Chinese cabbage (roots and leaves) at three periods (15, 35, and 60 days) under different treatments: no nitrogen (CK), 25 mg kg^−1^ NH_4_^+^-N (LA), 100 mg kg^−1^ NH_4_^+^-N (MA), 200 mg kg^−1^ NH_4_^+^-N (HA), 25 mg kg^−1^ NO_3_^−^-N (LN), 100 mg kg^−1^ NO_3_^−^-N (MN), and 200 mg kg^−1^ (HN). Errors bars represent standard deviations of triplicate samples. Different lowercase letters above the bars indicated significant differences among treatments at *p* < 0.05.

Nitrogen application also impact the ARG and MGE accumulation to vegetables. In the early growing stage of Chinese cabbage (15 days), application of NH_4_^+^-N and NO_3_^−^-N generally decreased *blaTEM-1* abundance but increased *sul1* and *tnpA-4* abundances in roots as compared to the control treatment. However, with Chinese cabbage growing (60 day), in general, the nitrogen treatment enhanced the accumulation of *blaTEM-1* and *sul1* to roots but inhibited that of *tnpA-4*. As shown in Figure 3, *blaTEM-1* abundances increased from 15 to 60 days under NH_4_^+^-N and NO_3_^−^-N treatments. But under control treatment, the *blaTEM-1* abundances decreased by 16% from 15 to 60 days. On the contrary, for *tnpA-4*, although at 15 days their abundances in nitrogen treatments were higher than that in control group, with vegetable growing to 60 days, the abundance in control group turned to be higher than those under the nitrogen treatments, suggesting *tnpA-4* accumulation in roots were inhibited by nitrogen. This trend showed a similar pattern to that of the abundances of *blaTEM-1*, *sul1 and tnpA-4* in soil under the nitrogen application (i.e., *blaTEM-1* and *sul1* abundances were increased and *tnpA* abundance was decreased at 60 days; Figure 2). Cerqueira et al. (2019a) also discovered that the abundance of ARGs in the roots of legume plants was highly associated with that in the soil. For ARGs in leaves, as compared the ARG abundances at 15 days with those at 60 days, generally, nitrogen application promoted *blaTEM-1* accumulation, and had no obvious effect on *sul1* and *tnpA-4* accumulation. For example, under the control treatment, the abundance of *blaTEM-1* at 15 days in the leaves was very closed to that at 60 days, which means the accumulation of this gene to the vegetable leave is limited. However, under the treatment of nitrogen, the abundance of *blaTEM-1* increased up to 80.44-fold higher from 15 to 60 days, indicating a stronger accumulation of this gene into Chinese cabbage leaves under nitrogen application. Similar to that on soil ARGs, we also compared the differences in the influencing degrees of NH_4_^+^-N and NO_3_^−^-N on ARG abundances in vegetables at 60 days. It was found that the differences in the regulating effects between NH_4_^+^-N and NO_3_^−^-N appeared lack of regularity, suggesting the nitrogen forms were no longer a crucial influencing factor on ARG abundances in vegetables. It is known that most vegetables prefer to utilize NO_3_^−^-N than NH_4_^+^-N (Zhang et al., 2016) and thus would adsorb more NO_3_^−^-N from soil compared to NH_4_^+^-N. The higher level of NO_3_^−^-N in vegetable might offset the stronger regulation of NH_4_^+^-N on the soil-derived ARB inside the Chinese cabbage, which might make the nitrogen forms no longer a crucial influencing factor.

Overall, the bacterial community composition and HGT majorly control the ARG distribution in various environmental niches (Barlow, 2009; Forsberg et al., 2014). According to a previous study, nitrogen application could alter the bacterial community structure (Fierer et al., 2012), which might be a mechanism accounting for the regulation of nitrogen on ARGs in soil and vegetable in this study. Besides that, the MGE *tnpA-4* abundances were also influenced by nitrogen application to certain extents, implying the HGT process affected by nitrogen application should be another mechanism.

### Distribution Patterns of ARGs and the MGE in Soil–Vegetable System

Although nitrogen application influenced the ARG and MGE abundances in vegetables to various degrees, it could not influence their distribution patterns (i.e., preference to either leaf or root tissues) in Chinese cabbage. As shown in Figure 1, different ARGs had different distribution patterns in vegetables under control treatment. For example, as the vegetables grew by 60 days, a large amount of *blaTEM-1* accumulated in the leaves, and its abundance was 3.38 times higher than that in the roots. But for *tnpA-4* and *sul1*, they were enriched in the roots. Particularly for *tnpA-4*, its abundance in roots was even 121.87 times higher than that in leaves. It is reported that the reason for the accumulation of *tnpA-4* in roots might be that this gene normally acts as a MGE (Cerqueira et al., 2019b), and the rhizosphere is the hotspot of HGT (Zhang et al., 2019). Previous studies have shown that the selective enrichment of foreign ARB in plant tissues may be the cause of different preferences of ARGs. For example, *Bacteroidetes* in rhizosphere could migrate to plant roots but rarely could further migrate to the leaves (Guo et al., 2021). Interestingly, after nitrogen application, be it in NH_4_^+^-N or NO_3_^−^-N forms, or even at low or high levels, could not alter the distribution patterns of these ARGs. As shown in Figure 3, for the LA, MA, HA, LN, MN, and HN treatments, *blaTEM-1* always preferred being in the leaves than in the roots. Previous studies also indicated a similar preference for *blaTEM-1* in plant leaves (Cerqueira et al., 2019a). *TnpA-4* and *sul1* were still enriched in vegetable roots (Figure 3). For each ARG, their endophyte host commonly has a relatively fixed ecological niche (Karmakar et al., 2019), which might not be easily changed by external factors such as nitrogen application.

### Bioaccumulation Factors and Transfer Factors

**Figure 4:**
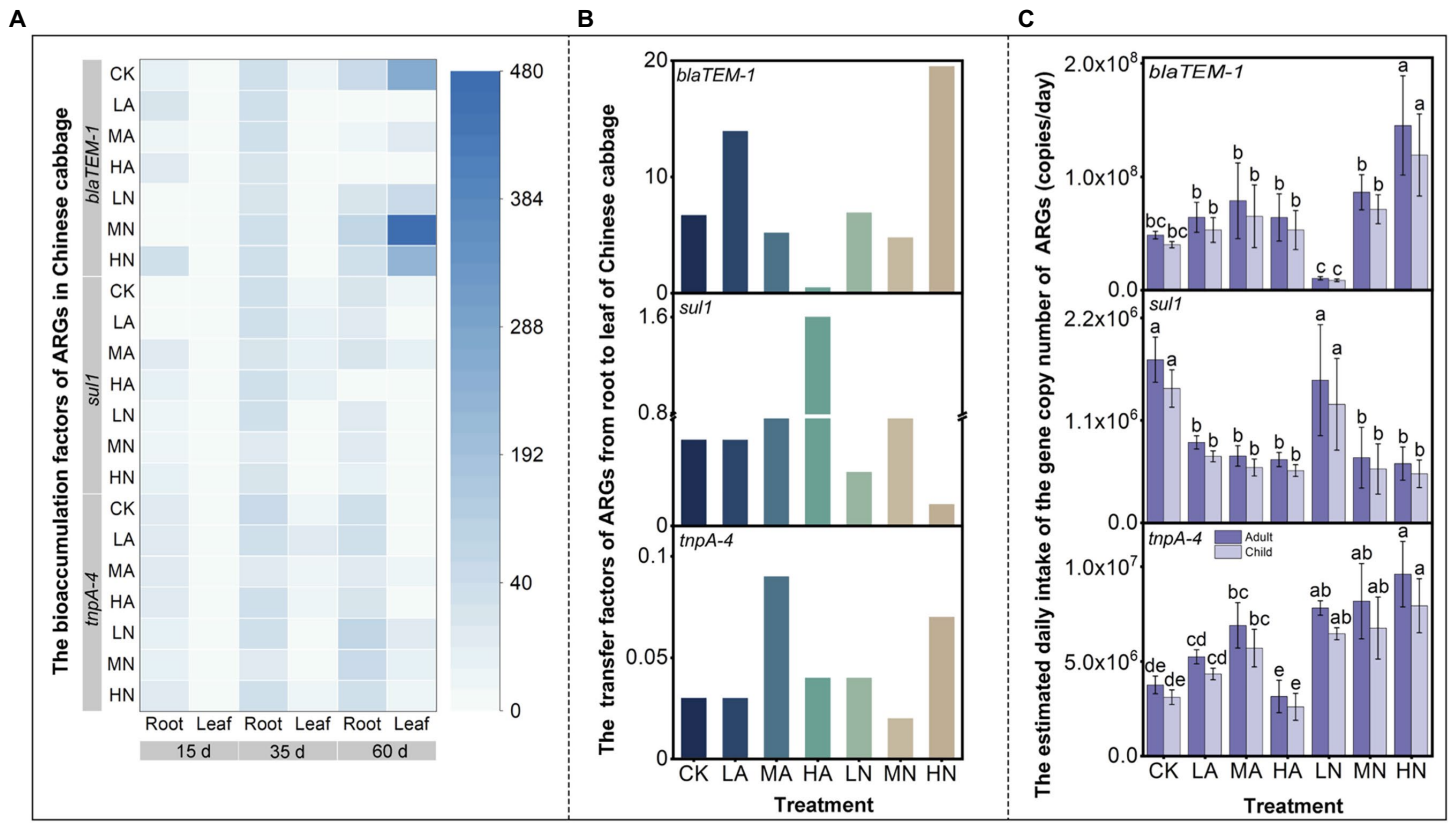
**(A)** Heatmap shows the bioaccumulation factors (BAF) of ARGs in the roots and leaves of Chinese cabbage. The color of white to blue represents the BAF values of the corresponding ARGs in different treatments and times. **(B)** The transfer factors (TF) of ARGs from roots to leaves at 60 days. **(C)** The estimated daily intake (EI) of ARGs by eating matured Chinese cabbage leaves (at 60 days). Different lowercase letters above the bars indicated significant differences among treatments at *p* < 0.05.

In our study, BAF was analyzed to determine the ARG enrichment capacity in different vegetable parts. The TF was also calculated to examine the transfer capacity of ARGs from roots to leaves. In Figure 4A, the blue colors in the root column were darker than those in the leaf column, illustrating the ARG enrichment capacities in roots were higher than those in leaves for both the control and nitrogen treatments (except for *blaTEM-1* gene at 60 days). This was in accordance with previous studies reported that rhizosphere is the main point of ARG migration from soil to plant (Van Elsas et al., 2003). Microbes played as an important carrier for ARGs to migrate into plants (Cordovez et al., 2019). They first move along the rhizosphere to the root surface and then colonize inside the root (Edwards et al., 2015). Therefore, root plays an indispensable role in the transfer of ARGs in the soil–vegetable system. However, for the *blaTEM-1* gene at 60 days, which was a special case, their enrichment capacities in leaves were higher than roots. As shown in Figure 4B, without or with nitrogen application (except for HA), the TFs of *blaTEM-1* gene at 60 days were significantly higher than those of *sul1* and *tnpA-4*. For example, for the control treatment, the TF of the *blaTEM-1* gene was close to 6.70, much higher than those for *sul1* and *tnpA-4*. This indicated that *blaTEM-1* had a higher transfer capacity (i.e., TFs) in Chinese cabbage, which made them easier to accumulate in leaves. Moreover, we found nitrogen application influenced the ARG transfer capacity in vegetable, but the influencing effect lacked regularity. For instance, the transportability of *blaTEM-1* was enhanced in LA and HN treatments, but those of *sul1* were enhanced in HA treatment, while *tnpA-4* was in MA and HN treatments. The influencing effect might not be intense, as the distribution patterns of ARGs in vegetables could not be altered by nitrogen application as described above.

### Estimated Daily Intake of ARGs in Human

We calculated the estimated daily intakes (EIs) of ARGs from the consumption of Chinese cabbage based on the study of Mei et al. (2021). The study investigated the ARG intake from eating the raw carrots, and reported that children and adults would intake 2.7 × 10^7^ and 3.2 × 10^7^ copies of ARGs per day, respectively. In this study, it was calculated that children and adults may intake up to 3.99 × 10^7^ and 4.83 × 10^7^ copies of *blaTEM-1*, 3.09 × 10^6^ and 3.74 × 10^6^ copies of *tnpA-4*, and 1.44 × 10^6^ and 1.75 × 10^6^ copies of *sul1* per day by consuming Chinese cabbage leaves under control treatment, respectively. The highest EI values of *blaTEM-1* may be attributed to the highest transfer capacity of this gene from root to leaf in Chinese cabbage (Figure 4B). Until now, there was no solid evidence demonstrating that consuming foods harboring ARGs would lead to an increase of ARGs in human body. However, HGT is recognized as a common event in human oral bacteria (Mira, 2008), and plasmids (a common vector of ARGs) was proven could get from mouth to gut of mammalian and persisted in gut for hours (Wilcks et al., 2004). These potentially indicated that vegetable-carrying ARGs particularly for those on MGEs had a probability to enter human body, which might transfer to the pathogens and thus threatened human health. Martínez et al. (2015) also pointed out that ARGs would pose a substantial risk for the dissemination of resistance if they reside on MGEs. In this study, we found NO_3_^−^-N application increased EI*_tnpA-4_* (i.e., the MGE) by 110%–160% (Figure 4C), which might increase the potential of ARG transfer from vegetables to human pathogens, enlarging health risks. Besides *tnpA-4*, application of NH_4_^+^-N increased EI*_blaTEM-1_* by 30%–60% (Figure 4C) but reduced EI*_sul1_* by 50%–60% (Figure 4C). This manifests that nitrogen application may differentially affect the intake of ARGs in human. Therefore, in future agricultural practice, it is suggested that nitrogen application should carefully refer to the ARG types in soil.

## Conclusion

The results in this study provided a new understanding on ARG distribution in soil-vegetable system under the agronomic measures of nitrogen application. For those genes that could transfer from soil to vegetables (*blaTEM-1*, *sul1*, and *tnpA-4*), nitrogen application could not change their distribution patterns in vegetables but could regulate their abundance. Under most circumstances, ARGs preferred being enriched in the roots of Chinese cabbage. When human consumed the Chinese cabbage leaves, the effect of nitrogen application on ARG intake varies with ARG types. These results are helpful in understanding the distribution and transfer of ARGs in the soil-vegetable system and provide data support for the health risk assessment of ARGs in future.

## Data Availability Statement

The original contributions presented in the study are included in the article/Supplementary Material, and further inquiries can be directed to the corresponding author.

## Author Contributions

TW, SS, YG, and XH designed the study. TW and SS conducted the experiments and drafted the manuscript. YX, MW, EO, GV, and XH proofread the manuscript. All authors contributed to the article and approved the submitted version.

## Funding

This work was funded by the National Natural Science Foundation of China (42107024 and 41925029), the Natural Science Foundation of Jiangsu Province of China (BK20210413), and the National Science Foundation for Postdoctoral Scientists of China (2021M691609).

## Conflict of Interest

The authors declare that the research was conducted in the absence of any commercial or financial relationships that could be construed as a potential conflict of interest.

## Publisher’s Note

All claims expressed in this article are solely those of the authors and do not necessarily represent those of their affiliated organizations, or those of the publisher, the editors and the reviewers. Any product that may be evaluated in this article, or claim that may be made by its manufacturer, is not guaranteed or endorsed by the publisher.
