# Supplementary material for: Nitrogen Regulates the Distribution of Antibiotic Resistance Genes in the Soil–Vegetable System
Source: Front Microbiol. 2022 Mar 14;13:848750. doi: 10.3389/fmicb.2022.848750 (PMC8964294; doi:10.3389/fmicb.2022.848750)
Supplement: Supplementary file 1 [file Data_Sheet_1.docx]

***Supplementary material for：***

**Nitrogen Regulates the Distribution of Antibiotic Resistance Genes in the Soil-Vegetable System**

Tingting Wang^1^, Silu Sun^1^, Yanxing Xu^1^, Michael Gatheru Waigi^1^, Emmanuel Stephen Odinga^1^, Galina K. Vasilyeva^2^, Yanzheng Gao^1^ and Xiaojie Hu^*1^

1. Institute of Organic Contaminant Control and Soil Remediation, College of Resources and Environmental Sciences, Nanjing Agricultural University, Nanjing 210095, P.R. China.

2. Institute of Physicochemical and Biological Problems in Soil Science, Russian Academy of Sciences, Pushchino, Moscow region, Russia

***Corresponding author:** Xiaojie Hu.

Address: Weigang Road 1, Nanjing 210095, China.

E-mail: huxiaojie@njau.edu.cn.

12 pages with 5 figures and 6 tables.

**
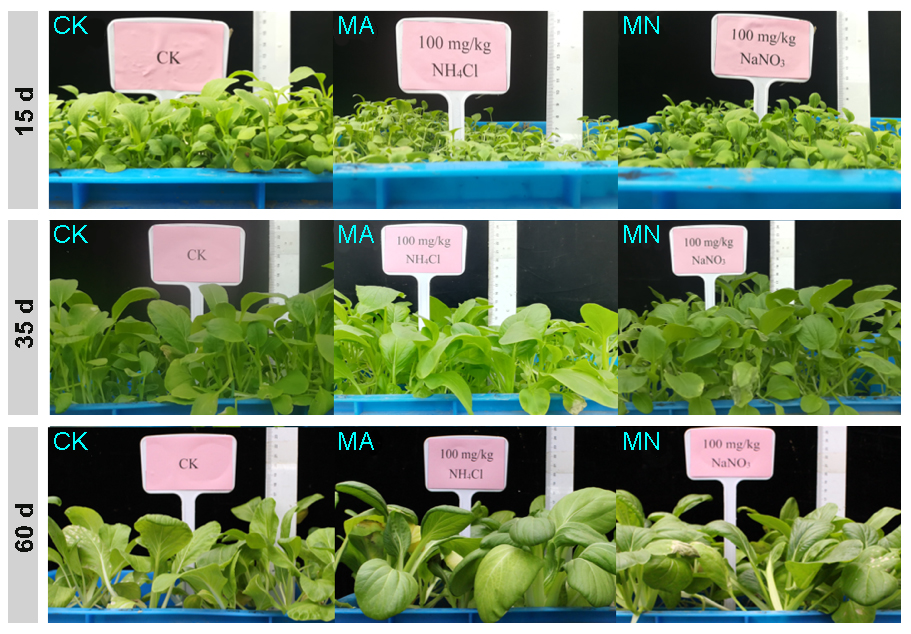
**

**Figure S1.** The photos of Chinese cabbages at 15 d, 35 d, and 60 d under CK, MA, and MN treatments.

**
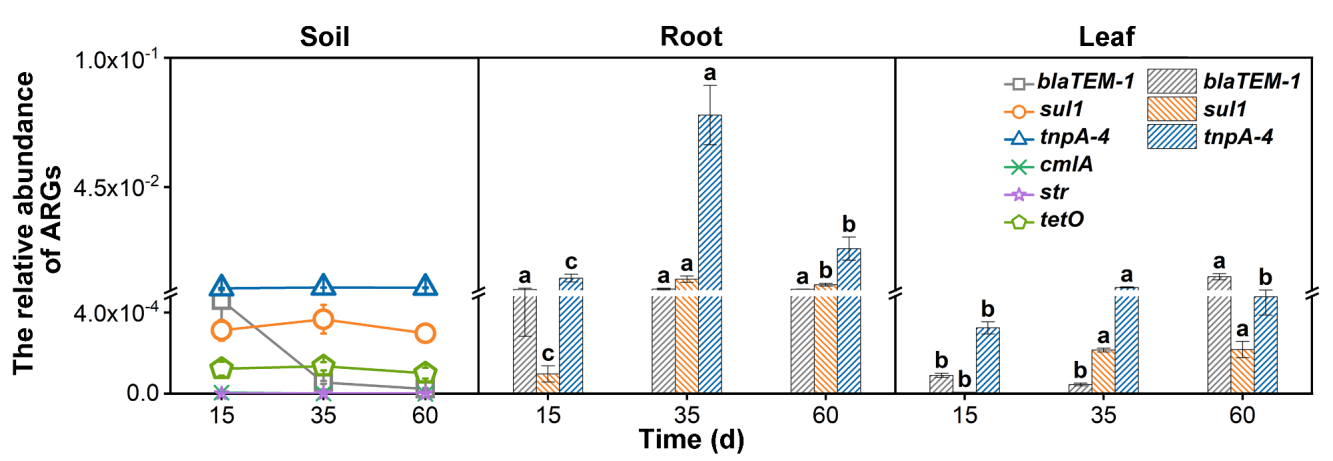
**

**Figure S2.** The relative abundance of ARGs in soil, root, and leaf at three growth periods (15 d, 35 d, and 60 d) with no nitrogen application (CK). Error bars represent standard deviations of triplicate samples. Different lowercase lettersabove the bars indicated significant differences among growth periods at *p* < 0.05.


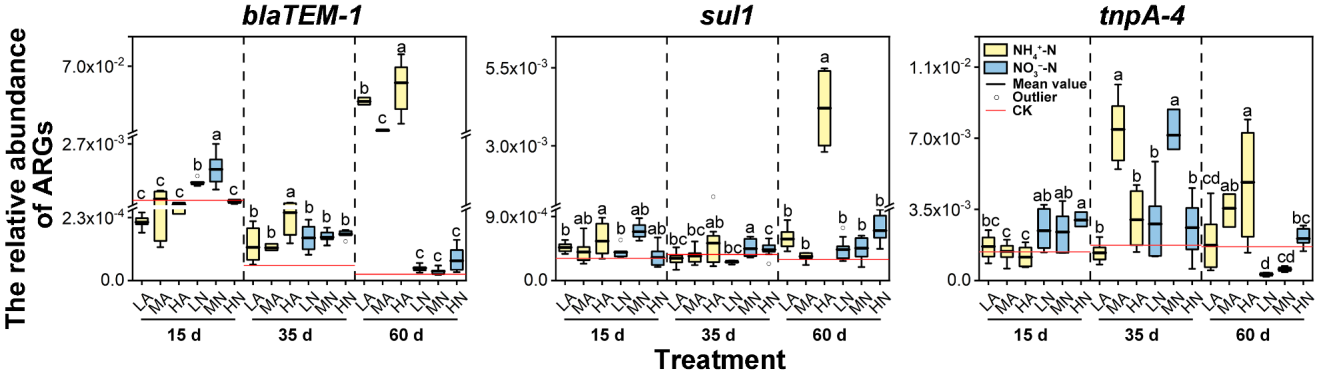


**Figure S3.** The relative abundance of the different ARGs (*blaTEM-1*, *sul1*, and *tnpA-4*) in the soil at three periods (15 d, 35 d, and 60 d) with nitrogen treatments: 25 mg kg^–1^ NH_4_^+^-N (LA), 100 mg kg^–1^ NH_4_^+^-N (MA), 200 mg kg^–1^ NH_4_^+^-N (HA), 25 mg kg^–1^ NO_3_^–^-N (LN), 100 mg kg^–1^ NO_3_^–^-N (MN), and 200 mg kg^–1^ NO_3_^–^-N (HN). Error bars represent standard deviations of triplicate samples. Different lowercase letters above the bars indicated significant differences among treatments at *p* < 0.05.


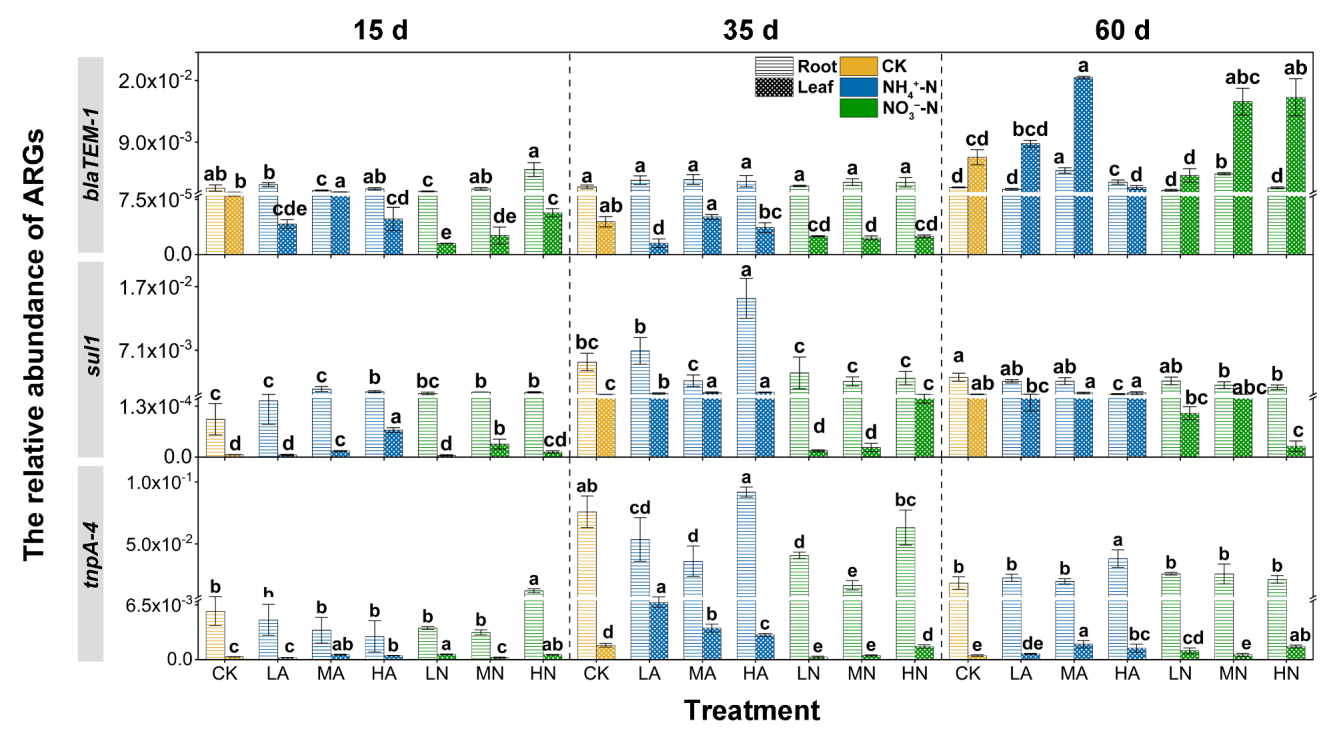


**Figure S4.** The relative abundance of the different ARGs (*blaTEM-1*, *sul1*, and *tnpA-4*) in Chinese cabbage (root and leaf) at three periods (15 d, 35 d, and 60 d) under different treatments: no nitrogen (CK), 25 mg kg^–1^ NH_4_^+^-N (LA), 100 mg kg^–1^ NH_4_^+^-N (MA), 200 mg kg^–1^ NH_4_^+^-N (HA), 25 mg kg^–1^ NO_3_^–^-N (LN), 100 mg kg^–1^ NO_3_^–^-N (MN), and 200 mg kg^–1^ NO_3_^–^-N (HN). Error bars represent standard deviations of triplicate samples. Different lowercase letters above the bars indicated significant differences among treatments at *p* < 0.05.


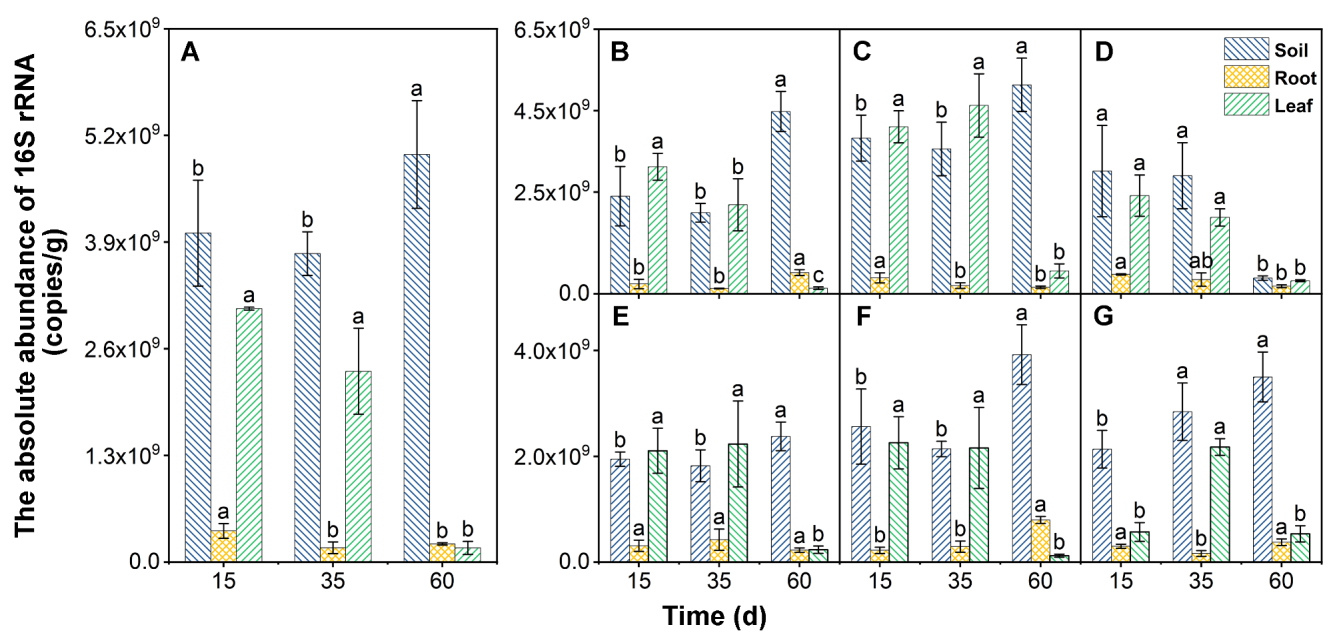


**Figure S5.** The absolute abundance of 16S rRNA in the soil-vegetable system under CK (A) and nitrogen application treatments (B: LA, C: MA, D: HA, E: LN, F: MN, G: HN). Different lowercase letters indicated significant differences (*p* < 0.05) in the abundance of ARGs among different growth periods.

**Table S1.** Physiochemicalproperties of sampling soil.

| pH | Organic matter  (g kg^-1^) | Moisture content  (%) | Total N  (g kg^-1^) | NO_3_^-^-N  (mg kg^-1^) | NH_4_^+^-N  (mg kg^-1^) |
| --- | --- | --- | --- | --- | --- |
| 6.6 | 21.8 | 27.5 | 2.3 | 115.5 | 0.94 |

**Table S2.** The background abundance of ARGs and 16S rRNA in soil.

|  | *blaTEM-1* | | *cmlA* | *str* | *sul1* | *tetO* | *tnpA-4* | 16S rRNA |
| --- | --- | --- | --- | --- | --- | --- | --- | --- |
| Absloute abundance (copies g^-1^) | | 1.1×10^7^ | 6.2×10^4^ | 1.7×10^4^ | 1.3×10^6^ | 1.8×10^5^ | 1.9×10^6^ | 5.9×10^9^ |

**Table S3.** PCR primer sequences for ARGs and 16S rRNA amplification.

| Gene | PCR primer sequence | Amplicon size (bp) | Annealing temperature (°C) | Gene categories |
| --- | --- | --- | --- | --- |
| *blaTEM-1* | F:AGCATCTTACGGATGGCATGA  R:TCCTCCGATCGTTGTCAGAAGT | 101 | 48 | *β*-lactam Resistance Gene |
| *sul1* | F: CACCGGAAACATCGCTGCA  R: AAGTTCCGCCGCAAGGCT | 158 | 57 | Sulfonamide Resistance Gene |
| *tnpA-4* | F: CCGATCACGGAAAGCTCAAG  R: GGCTCGCATGACTTCGAATC | 101 | 57 | IS6 Transposase |
| *cmlA* | F: GCCAGCAGTGCCGTTTAT  R: GGCCACCTCCCAGTAGAA | 158 | 54 | Chloramphenicol Resistance Gene |
| *str* | F: AATGAGTTTTGGAGTGTCTCAACGTA  R: AATCAAAACCCCTATTAAAGCCAAT | 148 | 53 | Aminoglycoside Resistance Gene |
| *tetO* | F: ATGTGGATACTACAACGCATGAGATT  R: TGCCTCCACATGATATTTTTCCT | 101 | 54 | Tetracycline Resistance Gene |
| 16S rRNA | F: ACTCCTACGGGAGGCAGCAG  R: GGACTACHVGGGTWTCTAAT | 468 | 60 | / |

**Table S4.** Reaction mixtures of qPCR.

| Gene | Template DNA (μL) | Forward Primer (μL) | Reverse Primer (μL) | AceQ qPCR SYBR Green  Master Mix (μL) | ddH_2_O (μL) |
| --- | --- | --- | --- | --- | --- |
| *blaTEM-1* | 1 | 0.4 | 0.4 | 10 | 8.2 |
| *sul1* | 1 | 0.4 | 0.4 | 10 | 8.2 |
| *tnpA-4* | 1 | 0.8 | 0.8 | 10 | 7.4 |
| *cmlA* | 1 | 0.2 | 0.2 | 10 | 8.6 |
| *str* | 1 | 0.2 | 0.2 | 10 | 8.6 |
| *tetO* | 1 | 0.8 | 0.8 | 10 | 7.4 |
| 16S rRNA | 1 | 0.5 | 0.5 | 10 | 8 |

**Table S5.** Time programs of qPCR.

| Gene | Pre-denaturation | | Denaturation | | Anneal | | Extension | | Number of cycles |
| --- | --- | --- | --- | --- | --- | --- | --- | --- | --- |
|  | T (℃) | Time (min) | T (℃) | Time (s) | T (℃) | Time (s) | T (℃) | Time (s) |  |
| *blaTEM-1* | 95 | 10 | 95 | 15 | 55 | 15 | 72 | 20 | 40 |
| *sul1* | 95 | 10 | 95 | 15 | 57 | 15 | 72 | 20 | 40 |
| *tnpA-4* | 95 | 10 | 95 | 15 | 55 | 15 | 72 | 20 | 40 |
| *cmlA* | 95 | 10 | 95 | 15 | 54 | 15 | 72 | 20 | 40 |
| *str* | 95 | 10 | 95 | 15 | 53 | 15 | 72 | 20 | 40 |
| *tetO* | 95 | 10 | 95 | 15 | 54 | 15 | 72 | 20 | 40 |
| 16S rRNA | 95 | 10 | 95 | 15 | 55 | 30 | 72 | 35 | 40 |

**Table S6.** The biomass of vegetables under different treatments.

| Treatments | Plant height (cm) | Fresh weight (g) |
| --- | --- | --- |
| CK | 28.2±1.4 | 4.31±0.21 |
| LA | 29.2±1.2 | 7.82±0.65 |
| MA | 28.6±0.8 | 7.04±0.52 |
| HA | 28.0±2.6 | 5.30±0.41 |
| LN | 28.2±1.7 | 5.13±0.59 |
| MN | 30.2±2.3 | 7.29±0.67 |
| HN | 28.6±2.1 | 6.17±0.19 |

Note: Data were Means ± SDs, n = 3.
